# Inflammatory Markers and Disease Progression in Moderate-Stage Retinitis Pigmentosa: Longitudinal Analysis from the Retinitis Pigmentosa Progression and Inflammatory Marker Registry Study

**DOI:** 10.1016/j.xops.2026.101381

**Published:** 2026-08-25

**Authors:** Sakurako Shimokawa, Kaho Yamamoto, Takahiro Hisai, Masatoshi Fukushima, Kohta Fujiwara, Yoshito Koyanagi, Shoji Notomi, Keijiro Ishikawa, Shoko Tsukamoto, Akie Hirata, Atsushi Takada, Fuyuka Miyahara, Naoki Nakashima, Yuko Kobayakawa, Go Mawatari, Masataka Ishizu, Naoki Toyama, Tomoko Kaida, Kazunori Miyata, Yasuhiro Ikeda, Koh-Hei Sonoda, Yusuke Murakami

**Affiliations:** 1 Department of Ophthalmology, Graduate School of Medical Sciences, Kyushu University, Fukuoka, Japan; 2 Department of Medical Informatics, Faculty of Medical Sciences, Kyushu University, Fukuoka, Japan; 3 Medical Information Center, Kyushu University Hospital, Fukuoka, Japan; 4 Center for Clinical and Translational Research, Kyushu University Hospital, Fukuoka, Japan; 5 Department of Ophthalmology, Faculty of Medicine, University of Miyazaki, Miyazaki, Japan; 6 Miyata Eye Hospital, Miyazaki, Japan

**Keywords:** Aqueous flare, Visual function, Disease progression, Prospective study, Retinitis pigmentosa

## Abstract

**Purpose:**

To investigate longitudinal changes in visual functional and structural measures and to evaluate whether inflammatory markers are associated with disease progression in patients with moderate-stage retinitis pigmentosa (RP).

**Design:**

Multicenter 24-month prospective cohort study (RP-PRIMARY [Retinitis Pigmentosa Progression and Inflammatory Marker Registry]); longitudinal analysis.

**Participants:**

Sixty-seven patients aged 20–70 years with typical moderate-stage RP; 66 patients completed 24-month follow-ups.

**Methods:**

ETDRS best-corrected visual acuity (BCVA), Humphrey Field Analyzer 10-2 perimetry, OCT, and fundus autofluorescence (AF) were assessed longitudinally. Aqueous flare and systemic inflammatory markers (interleukin 8 [IL-8], intermediate monocyte proportion, and high-sensitivity C-reactive protein [hs-CRP]) were measured annually. Longitudinal changes in functional and structural outcomes were evaluated using linear mixed-effects models. Associations between baseline inflammatory markers and disease progression were assessed using linear mixed-effects models, adjusted for age, sex, intraocular lens status, and baseline ellipsoid zone (EZ) vertical length.

**Main Outcome Measures:**

Annual rates of change in visual functional and structural measures and their associations with inflammatory markers.

**Results:**

Over the 24-month follow-up period, ETDRS BCVA, mean deviation, central retinal sensitivity, EZ length, central foveal thickness, and AF ring area all showed significant progression. Higher baseline aqueous flare was associated with faster decline in central 12-point mean retinal sensitivity and EZ vertical length. Higher baseline intermediate monocyte proportion and hs-CRP were associated with faster ETDRS BCVA decline. Conversely, higher aqueous flare and intermediate monocyte proportion were associated with slower AF ring constriction, while higher hs-CRP was associated with slower central foveal thinning. No significant longitudinal associations were observed for IL-8.

**Conclusions:**

Visual functional and structural measures demonstrated significant progression over 2 years in moderate-stage RP. Baseline aqueous flare, intermediate monocyte proportion, and hs-CRP were associated with disease progression, supporting their potential as exploratory biomarkers for identifying patients at risk of faster progression.

**Financial Disclosure(s):**

Proprietary or commercial disclosure may be found in the Footnotes and Disclosures at the end of this article.

Retinitis pigmentosa (RP), or rod–cone dystrophy, is one of the most prevalent inherited retinal dystrophies and is characterized by progressive degeneration of rod and cone photoreceptors.^1^^,^^2^ To date, more than 100 causative genes have been identified, yet no effective treatment is currently available.^3^, ^4^, ^5^, ^6^, ^7^, ^8^, ^9^, ^10^, ^11^, ^12^ Clinically, RP typically begins with night blindness, followed by concentric visual field loss and eventual central vision impairment, and it represents a major cause of legal visual impairment globally.^1^^,^^2^^,^^13^^,^^14^

Recent evidence suggests that neuroinflammation plays a critical role in modulating disease progression in RP.^15^, ^16^, ^17^, ^18^, ^19^, ^20^, ^21^, ^22^ Our prior retrospective studies, as well as those by others, have demonstrated significant associations between ocular inflammatory markers—such as aqueous flare—and central visual function and its decline.^19^^,^^23^, ^24^, ^25^, ^26^ Furthermore, systemic inflammatory markers, including serum high-sensitivity C-reactive protein (hs-CRP), interleukin-8 (IL-8), and circulating CD14^++^CD16^+^ inflammatory monocytes, have been associated with worse visual function.^27^, ^28^, ^29^

Retinal inflammation in RP involves various immune subsets, including homeostatic and disease-associated microglia, as well as peripherally recruited macrophages.^15^^,^^22^^,^^30^, ^31^, ^32^, ^33^, ^34^ Although the exact roles of each subset remain under investigation, recent evidence indicates that resident microglia can exert protective effects in retinal degeneration by removing apoptotic cells.^30^^,^^31^^,^^35^, ^36^, ^37^, ^38^, ^39^ In contrast, our previous study identified inflammatory monocytes and peripherally recruited macrophages as key mediators of cone degeneration in RP,^29^ consistent with other studies highlighting the detrimental role of monocytes/macrophages in retinal degeneration.^33^^,^^34^^,^^40^^,^^41^ Based on these findings, we are developing IV administered pitavastatin nanoparticles to target inflammatory monocytes and protect cone photoreceptors, with a clinical trial scheduled for 2026.

To design an efficient trial protocol and establish a natural history control, we launched the RP-PRIMARY (Retinitis Pigmentosa Progression and Inflammatory Marker Registry) study—a multicenter, 2-year prospective registry enrolling patients with moderate-stage RP who are eligible for future clinical trials.^42^ Patients were followed up every 3 months to collect comprehensive ophthalmologic and inflammatory data. The baseline data analysis of RP-PRIMARY study showed that aqueous flare was negatively associated with visual function, and implantation of an intraocular lens was most strongly associated with an increase in aqueous flare.^43^ In the present report, we provide the first longitudinal findings from the RP-PRIMARY cohort, investigating whether inflammatory markers—including aqueous flare, serum hs-CRP, IL-8, and circulating monocyte subsets—are associated with disease progression.

## Methods

### Ethics Statement

This study was approved by the institutional review boards of Kyushu University, University of Miyazaki, and Miyata Eye Hospital (approval number: #2023-27) and was conducted in accordance with the Declaration of Helsinki. Written informed consent was obtained from all participants before enrollment.

### Study Design and Protocol

This was the first 24-month longitudinal analysis conducted within the multicenter RP-PRIMARY cohort in Japan.^42^ Data were collected using standardized paper case report forms at baseline and follow-up visits. Demographic and background information included age, sex, age at disease onset, history of consanguineous marriage, causative gene, height, and body weight. Ocular history, including any complications, drug use, and past medical or surgical history, as well as the study eye, was recorded. The inheritance pattern was determined based on the causative genes. Systemic health information included comorbidities, supplement use, smoking status, and regular physical exercise.

Visual function and ocular imaging assessments consisted of ETDRS best-corrected visual acuity (BCVA), intraocular pressure, color fundus photography, Humphrey Field Analyzer 10-2 (HFA 10-2), mean deviation, central 12-point mean retinal sensitivity (RS Cent 4’), central 4-point mean retinal sensitivity,^44^ central foveal thickness (CFT), ellipsoid zone (EZ) horizontal and vertical length on OCT, and fundus autofluorescence (AF).

Blood biomarkers included hs-CRP, white blood cell count, leukocyte proportions (%neutrophils, %lymphocytes, and %monocytes), total cholesterol, high-density lipoprotein cholesterol and low-density lipoprotein cholesterol, estimated glomerular filtration rate, creatine kinase, serum IL-8, and circulating monocyte subsets classified as classical (CD14^++^CD16^–^), intermediate (CD14^++^CD16^+^), and nonclassical (CD14^+^CD16^++^).

ETDRS BCVA, HFA 10-2, OCT, and fundus AF were performed every 3 months, while aqueous flare measurement and blood tests, including monocyte flow cytometry, were conducted annually. Ocular and systemic data, including information on complications, medication use, surgical history, height, body weight, supplement use, smoking status, and physical activity, were updated annually. To ensure intercenter consistency, all measurement procedures were standardized using written standard operating procedures distributed to each participating site.

### Patient Enrollment

Participants were recruited between October 2021 and October 2022 at the three participating institutions. Typical RP was diagnosed according to the guidelines of a Japanese Ministry of Health, Labour and Welfare working group,^2^ as progressive symptoms with a history of night blindness, photophobia, visual field constriction and/or ring scotoma, and markedly reduced or nonrecordable a- and b-wave amplitudes on electroretinography testing, in addition to ophthalmoscopic findings (e.g., bone spicule-like pigment clumping, macular degeneration, and attenuation of retinal vessels).

The inclusion criteria included:

1. Diagnosis of typical RP (genetic testing not mandatory).
2. Age 20–70 years at consent.
3. Ability to understand the study purpose and provide consent.
4. Mean retinal sensitivity ≥10 dB within RS Cent 4’ on HFA 10-2.
5. CFT ≤250 μm on OCT.

The exclusion criteria included.

1. Anemia (Hb ≤8 g/dL).
2. Poor general condition (performance status ≥3).
3. Glaucoma or intraocular pressure ≥22 mmHg.
4. Uveitis or optic neuritis.
5. Retinal disorders unrelated to RP (e.g., retinal hemorrhage, retinal edema, and proliferative tissue).
6. Severe systemic illness.
7. Any other factor judged unsuitable by the investigators.

Inclusion criteria #4 and #5 were based on previous findings indicating nonlinearity of retinal structure–function relationship below 10 dB in RS Cent 4’ and faster progression in patients with thinner CFTs (≤252 μm, –0.78 dB/year), compared to those with thicker CFTs (>252 μm, –0.39 dB/year), respectively.^44^

For each participant, only one eye that fulfilled all eligibility criteria was included in the analysis. When both eyes were eligible, the eye without prior cataract surgery and without macular complications, such as epiretinal membrane or cystoid macular edema, was selected. If both eyes were comparable in these respects, the eye with the superior mean deviation on the HFA 10-2 test was selected.

### Sample Size Calculation

Our previous retrospective study demonstrated that RS Cent 4’ declined at –0.78 dB per year in patients with RP with CFT ≤252 μm.^44^ Based on this, the sample size was calculated with a presumed 30% treatment effect, yielding 170 patients (85 per group) for a two-arm study. After allowing for a 10% dropout rate, the RP-PRIMARY study set its target enrollment at 100 participants.

### Visual Acuity

Best-corrected visual acuity was measured with the ETDRS chart. Refraction was determined using an autorefractometer (ARK-1a) and refined to the most hyperopic correction. Testing was performed at 4 m; if ≤3 digits were identified on the top line, the test was repeated at 1 m with a +0.75 D lens. Scores were calculated as the number of correct digits at 4 m plus 30 (if ≥4 digits correct at 4 m), or as the sum of correct digits at 4 m and 1 m (if ≤3 at 4 m). If no digits were recognized, finger-counting, hand-motion, and light perception were sequentially assessed.

### Visual Field Testing

Visual field sensitivity was measured using the HFA 10-2 with the Swedish Interactive Threshold Algorithm Standard, prior to pharmacological dilation. Tests were conducted with appropriate lens correction. The stimulus parameters were: Goldmann III target, 200 ms duration, background luminance of 31.5 asb, and a maximum luminance of 10 000 asb. Tests were repeated if reliability criteria were not met (fixation loss >20%, false-positives >15%, and false-negatives >33%). Mean deviation was calculated from age-adjusted normative data, and central 4-point mean retinal sensitivity and RS Cent 4’ were computed as described previously.

### OCT Imaging and Structural Parameters

Retinal structure was assessed using spectral-domain OCT (SD-OCT; SPECTRALIS, Heidelberg Engineering). Horizontal and vertical B-scans were averaged over 50 frames. Central foveal thickness was measured using Heidelberg’s automated map with manual verification; when a discrepancy greater than 10 μm was observed between the center and minimum values, manual caliper measurements were performed. Ellipsoid zone length was defined as the distance between the disruption points of the EZ along the retinal pigment epithelium and was manually measured using a straight line tool; if the EZ was absent, the length was recorded as 0 mm. Autofluorescence ring area was assessed on AF images acquired with a 55° lens. The outer edge of the AF ring was manually traced, and values were recorded as 0 mm^2^ when central normal AF was absent or measured to the image boundary when the ring exceeded the imaging field. An indistinct hyper-AF ring area was not considered an exclusion criterion. All results were assessed by two independent examiners. Measurements of EZ length and AF ring area were performed by an experienced grader and independently reviewed by a retinal specialist. In cases of disagreement or when image interpretation was difficult, the final assessment was determined by consensus.

### Aqueous Flare Measurement

Aqueous flare was measured using a Kowa FM-600 laser flare photometer. After mydriasis using 0.5% tropicamide and 0.5% phenylephrine hydrochloride, measurements were taken under standardized conditions. Participants were aligned using chin and forehead rests, with fixation guided by an external target. Laser beams were aligned bilaterally outside the alignment marks, and scanning was initiated upon focus confirmation. Five scans were obtained per eye; outliers were excluded using the Smirnov–Grubbs test (significance level 5%). The average of three or more valid readings was used for analysis.

### Blood Sampling and Cytokine Testing

Routine blood parameters were tested at each local institution. For IL-8 and monocyte subset analyses, blood samples were processed within 1 h of collection, stored at –80 °C, and subsequently shipped for measurement of serum IL-8 and CD14^++^CD16^+^ intermediate monocyte (%) to Kyushu University. Serum IL-8 levels were quantified in duplicate using a multiplex ELISA system (Quansys Biosciences); samples with a coefficient of variation greater than 10% were retested. Monocyte subsets were analyzed by flow cytometry (CytoFLEX S; Beckman Coulter) using antibodies against CD2, CD14, CD16, CD19, CD56, and human leukocyte antigen-DR. Data were analyzed with FlowJo software, version 10.9.0 (BD Biosciences), and monocytes were classified into classical (CD14^++^CD16^–^), intermediate (CD14^++^CD16^+^), and nonclassical (CD14^+^CD16^++^) subsets.

### Statistical Analyses

Longitudinal changes in visual functional and structural outcomes over the 24-month follow-up period were evaluated using linear mixed-effects models with time as a fixed effect and subject-specific random intercepts. Differences in annual rates of change among genotype groups (*EYS*, *USH2A*, and Others) were assessed using the Kruskal–Wallis test (two-sided). When a significant overall group effect was detected, pairwise comparisons were performed using the Dunn post hoc test with Bonferroni adjustment for multiple comparisons. To evaluate associations between baseline inflammatory markers and disease progression, separate linear mixed-effects models were constructed for each outcome and inflammatory marker. The inflammatory markers evaluated were aqueous flare, serum IL-8, intermediate monocyte proportion, and hs-CRP. Each model included month, baseline inflammatory marker level, and their interaction term (Month × Marker) as fixed effects, together with age, sex, intraocular lens, and baseline EZ vertical length as covariates. These covariates were selected based on our previous baseline study, in which intraocular lens status was associated with aqueous flare levels and baseline disease severity.^43^ Participant-specific random intercepts were included to account for repeated measurements. The β coefficient represents the estimated effect of the Month × Marker interaction term and indicates the difference in the rate of longitudinal change according to baseline inflammatory marker level. Because inflammatory markers showed right-skewed distributions, aqueous flare, IL-8, intermediate monocyte proportion, hs-CRP, and EZ vertical length were natural log-transformed before analysis. Sensitivity analyses were additionally performed. Sensitivity model 1 further adjusted for cystoid macular edema and epiretinal membrane. Sensitivity model 2 further adjusted for genotype group (*EYS*, *USH2A*, and Others). To account for multiple testing, false discovery rate correction was performed using the Benjamini–Hochberg procedure, and both *P* values and false discovery rate–adjusted q values were reported. A two-sided *P* value <0.05 was considered statistically significant. All analyses were performed using R version 4.4.2 (R Foundation for Statistical Computing).

## Results

### Participants

A total of 80 participants provided written informed consent. Thirteen participants were excluded because they did not meet the eligibility criteria, leaving 67 participants enrolled in the RP-PRIMARY study (Fig 1). Although the planned sample size was 100 participants, enrollment was limited due to restrictions related to the COVID-19 pandemic. One participant was lost to follow-up after the baseline assessment because of relocation. Accordingly, all 67 participants were included in the baseline analyses, and 66 participants were included in the longitudinal analyses. 26 (38.8%) had both eyes meeting all eligibility criteria, and the eye with the better baseline HFA 10-2 mean deviation was selected. The most common reasons for fellow-eye exclusion were macular pathology (n = 25), previous cataract surgery (n = 12), RS Cent 4′ <10 dB (n = 9), CFT >250 μm (n = 6), and previous vitrectomy (n = 4); these categories were not mutually exclusive. Baseline demographic and genetic characteristics are summarized in Table 1. Baseline ocular characteristics were summarized in Table 2. At baseline, 45 of 66 eyes (68.2%) had an AF ring area of 0 mm^2^, and one eye (1.5%) was considered to have reached the image boundary. At 24 months, 47 of 66 eyes (71.2%) had an AF ring area of 0 mm^2^, and one eye (1.5%) was considered to have reached the image boundary. Baseline distributions of inflammatory markers are summarized in Supplementary Table S1, available at www.ophthalmologyscience.org.

**Figure 1:**
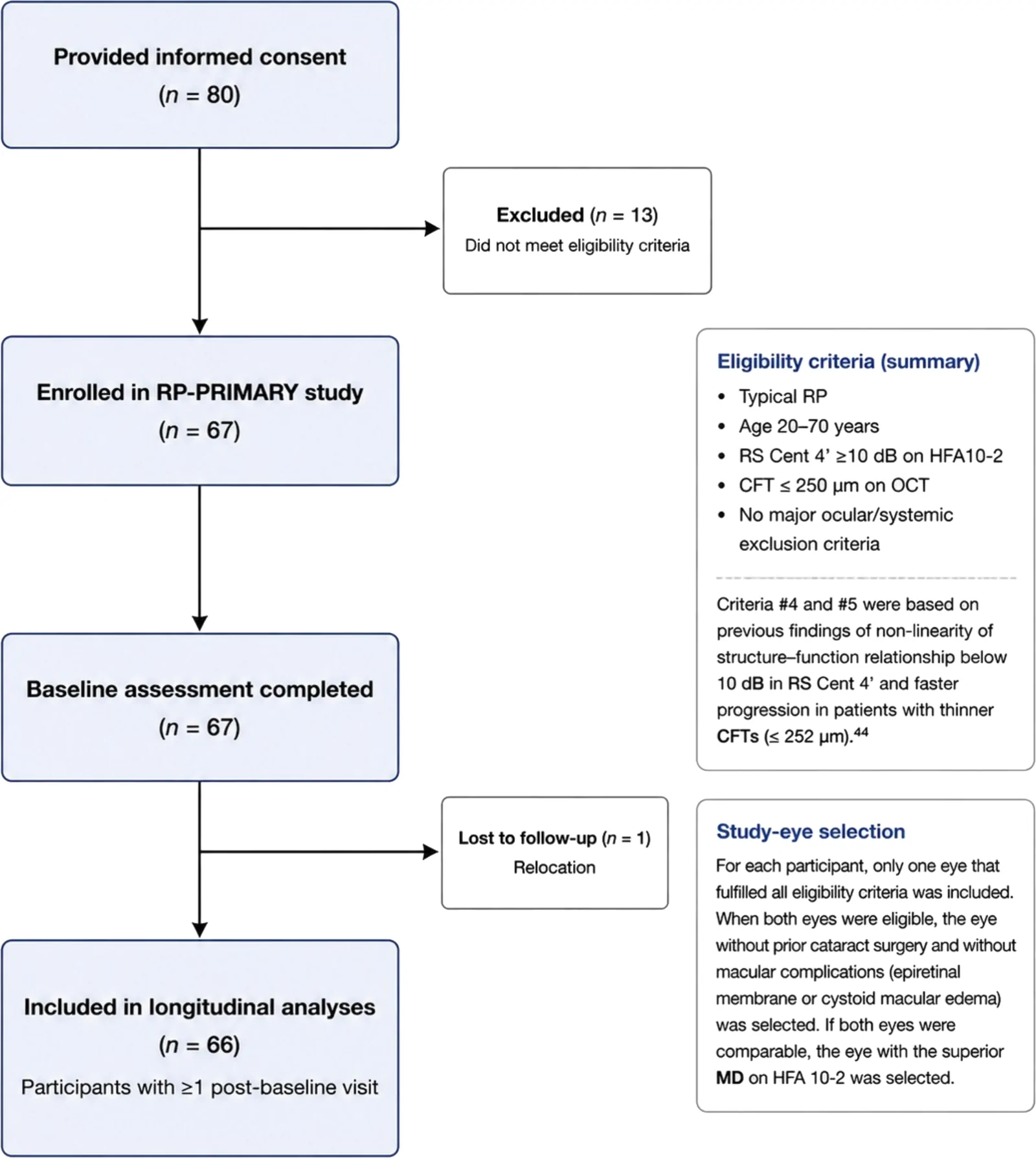
Flow diagram of participant recruitment and follow-up. Eighty participants provided informed consent, of whom 13 were excluded because they did not meet the eligibility criteria. Sixty-seven participants were enrolled in the RP-PRIMARY study. One participant was lost to follow-up because of relocation, leaving 66 participants for the longitudinal analyses. Eligibility criteria and study-eye selection are described in the Methods. RP = retinitis pigmentosa; RP-PRIMARY = Retinitis Pigmentosa Progression and Inflammatory Marker Registry; RS Cent 4′ = central 12-point mean retinal sensitivity; HFA = Humphrey Field Analyzer; CFT = central foveal thickness; MD = mean deviation.

**Table 1:** Patient Background of RP-PRIMARY Study

Characteristic	Value, No. of Patients = 67
Age	51.0 [43.0, 60.0]
Sex	
Male	25 (37%)
Female	42 (63%)
Onset age	27.0 [15.0, 40.0]
Disease duration	24.0 [10.0, 33.0]
Inheritance mode	
AD	4
Causative gene	*RHO* (n = 1), *SNRNP200* (n = 1), *TOPORS* (n = 1), *PROM1* (n = 1)
AR	26
Causative gene	*EYS* (n = 17), *USH2A* (n = 5), *ABCA4* (n = 1), *PDE6A* (n = 1), *PDE6B* (n = 2)
XL	1
Causative gene	*RPGR* (n = 1)
Not determined	28
Not tested	8
Ethnicity	Japanese 67 (100%)

AD = autosomal dominant; AR = autosomal recessive; RP-PRIMARY= Retinitis Pigmentosa Progression and Inflammatory Marker Registry; XL = X-linked.

Values are presented as median [Q1, Q3] or number (%). Disease duration was calculated as age at enrollment minus age at symptom onset. Inheritance modes included autosomal dominant, autosomal recessive, X-linked, sporadic, and unknown cases.

**Table 2:** Baseline Ocular Characteristics and Ocular Complications

Characteristic	Value, No. of Patients = 67
Baseline ocular characteristics	
ETDRS BCVA	74.0 [62.5, 79.5]
MD	–16.2 [-21.4, –13.2]
RS Cent 4′	24.4 [19.6, 27.6]
RS Cent 1′	28.2 [21.9, 31.6]
Central foveal thickness	186.0 [154.0, 216.5]
EZ vertical length	1 [0, 2]
EZ horizontal length	1 [0, 2]
AF ring area	0.00 [0.00, 3.11]
Ocular complications and surgical history	
Pseudophakic eye	14 (20.9%)
Cataract	28 (41.8%)
Epiretinal membrane	6 (9.0%)
Cystoid macular edema	3 (4.5%)
Previous vitrectomy	3 (4.5%)

AF = autofluorescence; BCVA = best-corrected visual acuity; EZ = ellipsoid zone; MD = mean deviation; RS Cent 4′ = central 12-point mean retinal sensitivity; RS Cent 1′ = central 4-point mean retinal sensitivity.

Values are presented as median [interquartile range] or number of participants (%). Outcome measures: BCVA (letters); MD (dB); RS Cent 4′ dB; RS Cent 1′ dB; EZ length (mm); AF ring area (mm^2^).

### Longitudinal Changes in Functional and Structural Measures

Over the 24-month follow-up period, annual rates of change for each functional and structural parameter were estimated using linear mixed-effects models (Table 3). ETDRS visual acuity significantly declined by –1.18 letters/year (95% confidence interval [CI], –1.69 to –0.67; *P* < 0.001). Mean deviation also showed a significant decrease of –0.24 dB/year (95% CI, –0.38 to –0.09; *P* = 0.002). For retinal sensitivity, RS Cent 4′ declined by –0.52 dB/year (95% CI, –0.71 to –0.33; *P* < 0.001), and central 4-point mean retinal sensitivity declined by –0.41 dB/year (95% CI, –0.65 to –0.16; *P* = 0.001). Among structural measures, log-transformed EZ vertical length [ln(mm + 1)/year] and EZ horizontal length [ln(mm + 1)/year] decreased by –0.05/year (95% CI, –0.06 to –0.04; *P* < 0.001) and –0.06/year (95% CI, –0.07 to –0.05; *P* < 0.001), respectively. On the original scale, the corresponding annual changes were –95.3 μm/year (95% CI, –111.4 to –79.1; *P* < 0.001) and –98.2 μm/year (95% CI, –115.7 to –80.6; *P* < 0.001), respectively. Central foveal thickness decreased by –2.19 μm/year (95% CI, –3.03 to –1.36; *P* < 0.001). Log-transformed AF ring area [ln(mm^2^ + 1)/year] also decreased significantly, with an annual slope of –0.07/year (95% CI, –0.09 to –0.04; *P* < 0.001). Exploratory genotype subgroup analyses showed differences in the progression of EZ length among genotype groups (Kruskal–Wallis test, *P* = 0.032). Pairwise comparisons indicated significantly greater log-transformed EZ vertical length [ln(mm + 1)/year] shortening in the *EYS* group (Dunn post hoc test, *P* = 0.01) (Fig 2). Changes in ocular conditions, medications, and surgical history during follow-up were infrequent (Supplementary Table S2, available at www.ophthalmologyscience.org).

**Table 3:** Slope of Visual Functional and Structural Endpoints

Endpoint	Slope, 95% CI, No. of Patients = 67	*P* Value
ETDRS BCVA	–1.18 (–1.69 to –0.67)	**<0.001**
MD	–0.24 (–0.38 to –0.09)	**0.002**
RS Cent 4’	–0.52 (–0.71 to –0.33)	**<0.001**
RS Cent 1’	–0.41 (–0.65 to –0.16)	**0.001**
EZ vertical length	–0.05 (–0.06 to –0.04)	**<0.001**
EZ horizontal length	–0.06 (–0.07 to –0.05)	**<0.001**
CFT	–2.19 (–3.03 to –1.36)	**<0.001**
AF ring area	–0.07 (–0.09 to –0.04)	**<0.001**

AF = autofluorescence; BCVA = best-corrected visual acuity; EZ = ellipsoid zone; MD = mean deviation; RS Cent 4′ = central 12-point mean retinal sensitivity; RS Cent 1′ = central 4-point mean retinal sensitivity.

Slopes represent annualized rates of change estimated using linear mixed-effects models with time (months) as a fixed effect and subject-specific random intercepts. All available longitudinal measurements over the 24-month follow-up period were included. Outcome measures: ETDRS BCVA (letters/year); MD (dB/year); RS Cent 4′ dB/year; RS Cent 1′ dB/year; EZ vertical length [ln(mm + 1)/year]; EZ horizontal length [ln(mm + 1)/year]; central foveal thickness (μm/year); AF ring area [ln(mm^2^ + 1)/year].

Bold values indicate statistical significance (2-sided *P* < 0.05).

**Figure 2:**
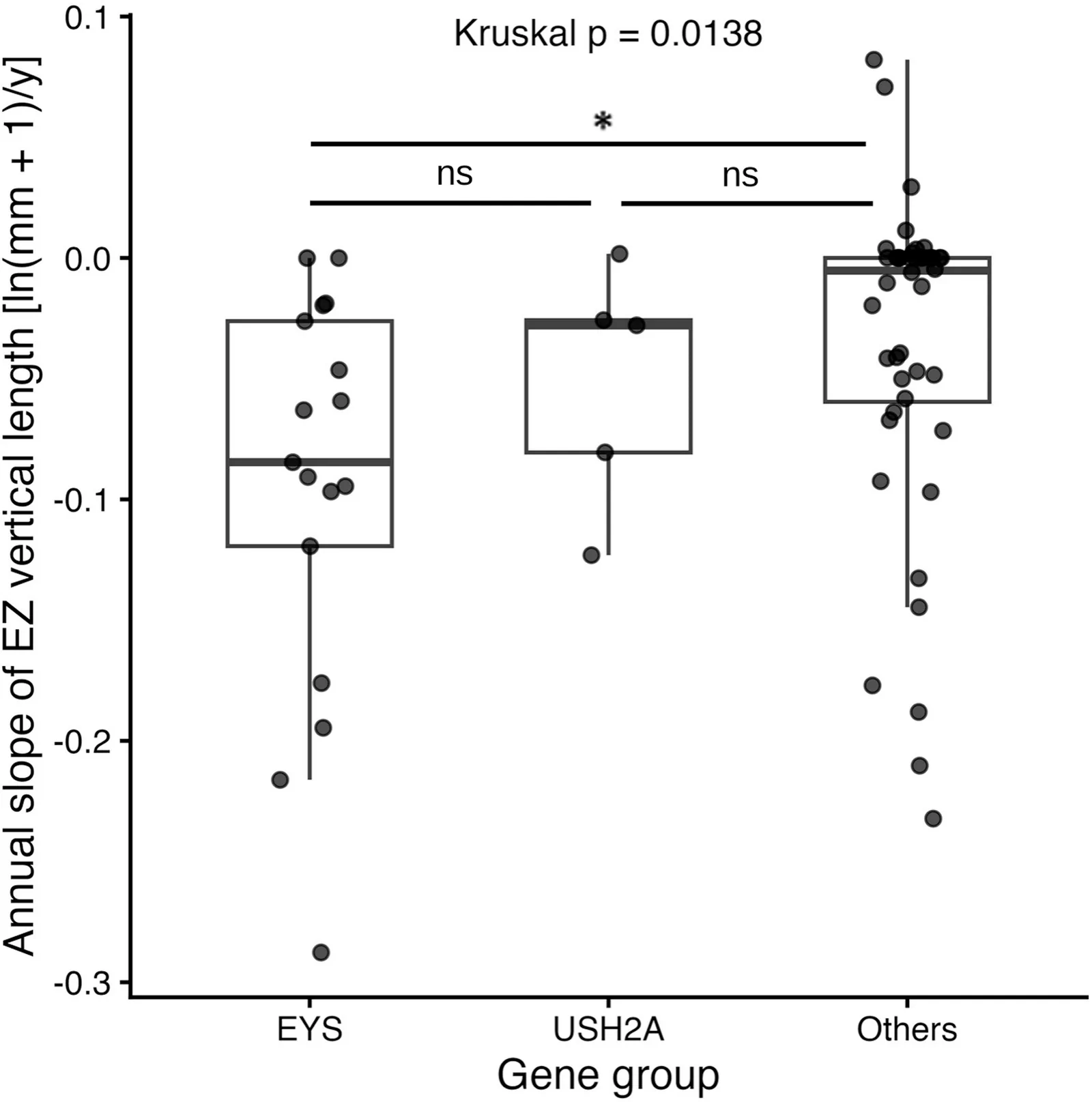
Comparison of annual EZ vertical length decline by genotype. Exploratory comparison of annual changes in log-transformed EZ vertical length among genotype groups. Annual rates of change in EZ vertical length were estimated for each subject using linear regression of log-transformed EZ length [ln(mm + 1)/y]. Box plots show the distribution of annual slopes in the *EYS*, *USH2A*, and Other groups. Boxes represent the IQR, center lines indicate medians, and whiskers extend to 1.5 × IQR. Points represent individual subjects. Group differences were assessed using the Kruskal–Wallis test (2-sided), followed by Dunn post hoc test for pairwise comparisons with adjustment for multiple testing. ∗Adjusted *P* < 0.05. EZ = ellipsoid zone; IQR = interquartile range.

### Association between Inflammatory Markers and Disease Progression

Associations between inflammatory markers and disease progression were evaluated using linear mixed-effects models. Higher baseline aqueous flare was significantly associated with faster decline in RS Cent 4′ (β = –0.040, q = 0.030) and log-transformed EZ vertical length [ln(mm + 1)/year] (β = –0.002, q = 0.016). Higher baseline intermediate monocyte proportion (β = –0.158, q = 0.018) and higher baseline hs-CRP (β = –0.138, q = 0.030) were also significantly associated with faster ETDRS BCVA decline. Conversely, higher baseline aqueous flare (β = 0.008, q = 0.002) and intermediate monocyte proportion (β = 0.008, q = 0.016) were associated with slower AF ring constriction, while higher baseline hs-CRP was associated with slower central foveal thinning (β = 0.261, q = 0.016). No significant associations were observed for IL-8 after false discovery rate correction (Fig 3, Supplementary Figure S1, Supplementary Table S3, available at www.ophthalmologyscience.org). The results remained largely unchanged after additional adjustment for cystoid macular edema and epiretinal membrane (Supplementary Table S4, available at www.ophthalmologyscience.org) or genotype group (Supplementary Table S5, available at www.ophthalmologyscience.org). Similarly, the results for vertical and horizontal EZ length remained largely unchanged after removing baseline log-transformed vertical EZ length as a covariate from the corresponding models (Supplementary Table S6, available at www.ophthalmologyscience.org). Assessment of linearity using an additional quadratic time term showed no significant deviation from linearity for any outcome over the 24-month follow-up period (all *P* > 0.35; Supplementary Table S7, available at www.ophthalmologyscience.org).

**Figure 3:**
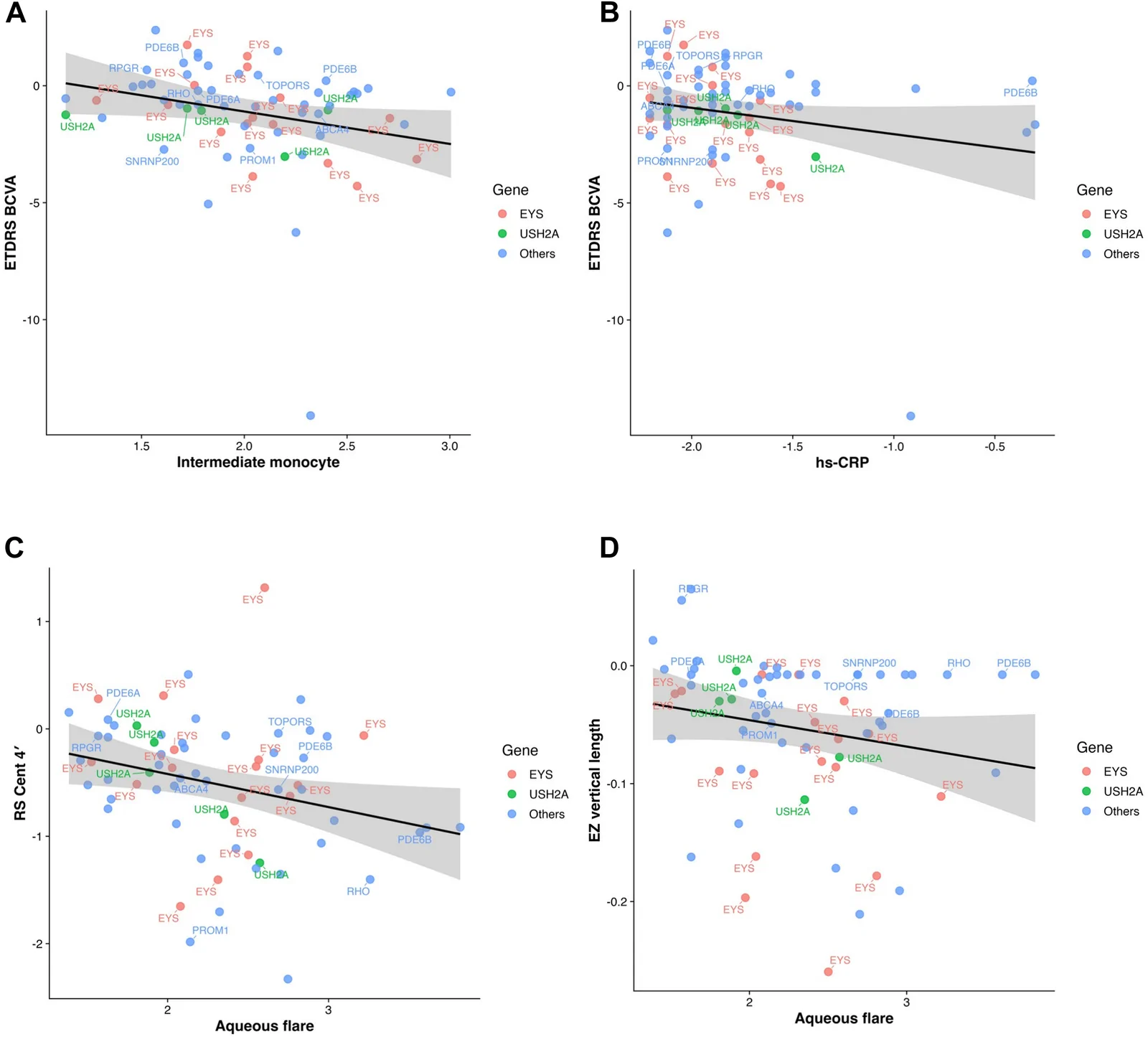
Associations between baseline inflammatory markers and RP progression. Scatter plots illustrate the associations between baseline inflammatory marker levels and individual annualized rates of change estimated using linear mixed-effects models. Significant associations are shown for (**A**) intermediate monocytes and ETDRS BCVA, (**B**) hs-CRP and ETDRS BCVA, (**C**) aqueous flare and central retinal sensitivity within RS Cent 4′, and (**D**) aqueous flare and EZ vertical length. Each point represents one participant. Regression lines with 95% confidence intervals are shown for visualization. ETDRS BCVA = ETDRS best-corrected visual acuity (letters/year); RS Cent 4′ = central 12-point mean retinal sensitivity (dB/year); EZ = ellipsoid zone [ln(mm + 1)/year]; hs-CRP = high-sensitivity C-reactive protein [ln(mg/dL + 0.1)]; aqueous flare [ln(pc/ms + 0.1)]; intermediate monocyte [ln(% + 0.1)]; RP = retinitis pigmentosa.

## Discussion

We demonstrated significant progression of visual functional and structural measures over 24 months in patients with moderate-stage RP. Longitudinal mixed-effects analyses further demonstrated significant associations between baseline aqueous flare, intermediate monocyte proportion, and hs-CRP and several measures of disease progression.

In patients with RP, the pattern and rate of disease progression have been challenging to characterize due to genetic heterogeneity and the typically slow, insidious course of degeneration. Our prospective longitudinal evaluation over 24 months provides evidence that ETDRS visual acuity, central retinal sensitivities, EZ length, and CFT decline measurably in moderate-stage RP, consistent with prior natural history studies.^44^, ^45^, ^46^, ^47^, ^48^, ^49^, ^50^, ^51^, ^52^ Exploratory analyses suggested differences in the rate of EZ progression among genetic subgroups. While functional progression rates did not diverge markedly across genes, structural decline in EZ length was more pronounced in *EYS* carriers compared with other genotypes. Although the findings should be interpreted cautiously because of the small sample size and the mixed genetic background in the Others group, this aligns with previous data suggesting genotype-dependent natural history patterns in RP.^49^^,^^53^, ^54^, ^55^ Such gene-specific progression differences have implications for clinical trial design and may argue for stratified randomization or genotype-specific endpoints in future therapeutic studies.

Another important dimension to consider in RP progression is the role of blood ocular barrier disruption and neuroinflammation. Although RP is primarily a genetic photoreceptor dystrophy, recent evidence supports that neuroinflammation and blood–retina barrier disruption may influence disease progression.^16^^,^^17^^,^^28^^,^^29^^,^^56^^,^^57^ Aqueous flare reflects blood–ocular barrier disruption as well as intraocular inflammation^58^^,^^59^ and has been associated with worse visual function in RP.^25^^,^^26^ Recent evidence further suggests that blood–retina barrier disruption may independently contribute to retinal degeneration.^60^ Our longitudinal analyses also support a link between systemic inflammatory activity and RP progression. Intermediate monocytes were associated with faster ETDRS BCVA decline, consistent with our previous findings implicating monocyte-derived macrophages in cone degeneration.^29^ Higher hs-CRP levels, a marker of chronic low-grade systemic inflammation that has been associated with disease severity and progression in several neurodegenerative disorders^61^, ^62^, ^63^ were also associated with faster ETDRS BCVA decline, further supporting a role of systemic inflammation in disease progression. Unexpectedly, higher aqueous flare and intermediate monocyte levels were associated with slower AF ring constriction, and higher hs-CRP levels were associated with less central foveal thinning. These findings should be interpreted with caution. Many participants had low or nonmeasurable AF ring areas at baseline, limiting the measurable range for subsequent change and potentially influencing the estimated association. Furthermore, the association between hs-CRP and CFT may have been influenced by the small number of participants with relatively high hs-CRP levels, and hs-CRP may also be affected by various systemic factors. Further studies are warranted to elucidate the relationships among barrier dysfunction, inflammation, retinal structural changes, and visual decline in RP.

We evaluated central retinal sensitivity using the HFA 10-2 protocol in this study. Although microperimetry and full-field stimulus testing have been used in clinical trials for RP,^3^^,^^5^^,^^64^, ^65^, ^66^, ^67^, ^68^ HFA 10-2 is widely available and highly feasible for standardized multicenter studies in Japan. We previously demonstrated that HFA 10-2 performs comparably to microperimeter MP-3 in patients with moderate-stage RP, showing similar cross-sectional and longitudinal correlations with EZ length.^69^ Furthermore, HFA 10-2–derived central retinal sensitivity has been accepted as a clinical trial endpoint by the Japanese regulatory authority, supporting its translational relevance (NCT01786395).^68^^,^^70^

### Limitations

Several limitations should be acknowledged. First, the relatively modest sample size may limit the generalizability of the findings. Second, the observational design precludes causal inference. Third, the sample size precluded subgroup analyses according to genetic subtype. Finally, because the study included only Japanese patients, validation in larger, multiethnic cohorts is warranted.

## Conclusion

Aqueous flare, the proportion of intermediate monocytes, and hs-CRP may serve as potential exploratory biomarkers of disease progression in moderate-stage RP and help identify patients at risk of faster progression.

## Declaration of Generative AI and AI-Assisted Technologies in the Writing Process

During the preparation of this work, the authors used ChatGPT (OpenAI) to assist in language editing and refinement. After using this tool, the authors reviewed and edited the content as needed and take full responsibility for the content of the publication.
