# Supplementary material for: Inflammatory Markers and Disease Progression in Moderate-Stage Retinitis Pigmentosa: Longitudinal Analysis from the Retinitis Pigmentosa Progression and Inflammatory Marker Registry Study
Source: Ophthalmol Sci. 2026 Aug 25;6(11):101381. doi: 10.1016/j.xops.2026.101381 (PMC13634498; doi:10.1016/j.xops.2026.101381)
Supplement: Supplementary file 1 [file mmc1.pdf]

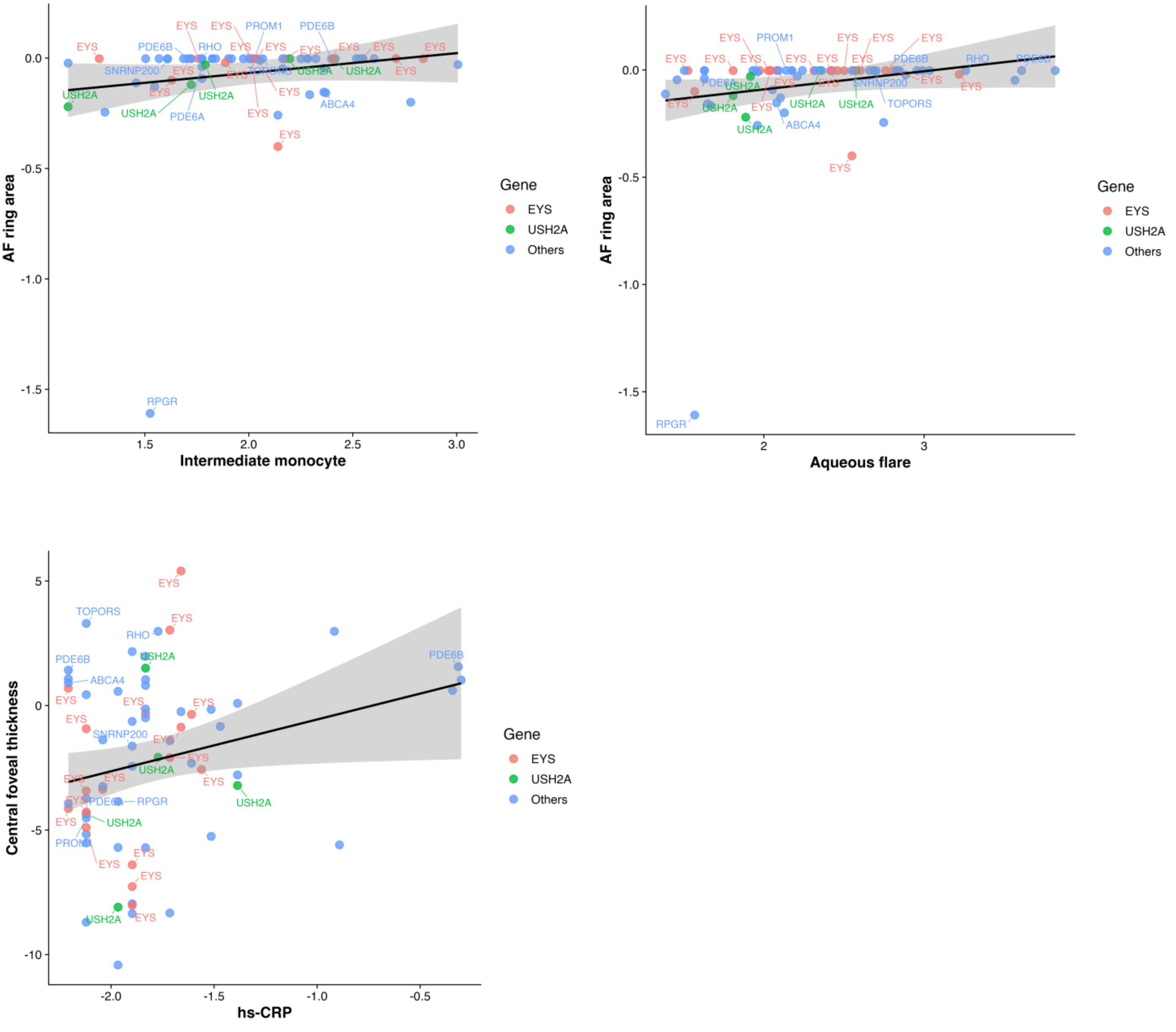

Supplementary Figure S1. Positive associations between inflammatory markers and RP progression

Scatter plots illustrating the unexpected positive associations observed between baseline inflammatory marker levels and individual annualized rates of change in structural outcomes. (A) Aqueous flare and AF ring area, (B) intermediate monocytes and AF ring area, and (C) hs-CRP and central foveal thickness. Each point represents one participant. Regression lines with 95% confidence intervals are shown for visualization only. AF = fundus autofluorescence; hs-CRP = high-sensitivity C-reactive protein [ln(mg/dL + 0.1)]; aqueous flare [ln(pc/ms + 0.1)]; intermediate monocyte [ln(% + 0.1)]; AF ring area [ln(mm² + 1)/year]; central foveal thickness (μm/year).
