# Supplementary material for: Inflammatory Markers and Disease Progression in Moderate-Stage Retinitis Pigmentosa: Longitudinal Analysis from the Retinitis Pigmentosa Progression and Inflammatory Marker Registry Study
Source: Ophthalmol Sci. 2026 Aug 25;6(11):101381. doi: 10.1016/j.xops.2026.101381 (PMC13634498; doi:10.1016/j.xops.2026.101381)
Supplement: Supplementary file 2 [file mmc2.pdf]

**Supplementary Table S1. Distribution of Baseline Inflammatory Markers**

| Inflammatory marker | No. of patients | Median | IQR | Range | No. of outliers |
|---|---|---|---|---|---|
| Aqueous flare | 67 | 9.00 | 6.85–14.60 | 3.90–45.40 | 3 |
| IL-8 | 67 | 12.00 | 9.20–15.35 | 4.20–41.10 | 4 |
| Intermediate monocyte | 66 | 7.40 | 5.55–10.02 | 3.00–20.10 | 2 |
| hs-CRP | 67 | 0.05 | 0.02–0.08 | 0.01–0.64 | 5 |

*Outliers were defined using the 1.5 × interquartile range (IQR) rule. Values are presented at baseline (Month 0). The sample size for inflammatory monocytes was reduced to n = 66 because of missing data. IL-8 = interleukin-8 (pg/mL); hs-CRP = high-sensitivity C-reactive protein (mg/dL).
