# Supplementary material for: Inflammatory Markers and Disease Progression in Moderate-Stage Retinitis Pigmentosa: Longitudinal Analysis from the Retinitis Pigmentosa Progression and Inflammatory Marker Registry Study
Source: Ophthalmol Sci. 2026 Aug 25;6(11):101381. doi: 10.1016/j.xops.2026.101381 (PMC13634498; doi:10.1016/j.xops.2026.101381)
Supplement: Supplementary file 3 [file mmc3.pdf]

Supplementary Table S2. New ocular conditions, medications, or surgical interventions during follow-up

Characteristic	No. of patients = 67
Cataract	5 (7.4%)
New IOL	0 (0%)
ERM	6 (8.8%)
CME	2 (2.9%)
Dorzolamide eye drops	2 (2.9%)
Unoprostone eye drops	1 (1.5%)
Other eye drops	0 (0%)
Other ocular history	0 (0%)
Vitrectomy	0 (0%)
Other ocular surgery	0 (0%)

* Values are presented as number (%). Values indicate the number of patients who changed from absent at baseline to present during the 24-month follow-up. Assessments were performed at baseline and at the 1-year and 2-year follow-up visits. IOL = intraocular lens; ERM = epiretinal membrane; CME = cystoid macular edema.
