# Supplementary material for: Inflammatory Markers and Disease Progression in Moderate-Stage Retinitis Pigmentosa: Longitudinal Analysis from the Retinitis Pigmentosa Progression and Inflammatory Marker Registry Study
Source: Ophthalmol Sci. 2026 Aug 25;6(11):101381. doi: 10.1016/j.xops.2026.101381 (PMC13634498; doi:10.1016/j.xops.2026.101381)
Supplement: Supplementary file 6 [file mmc6.pdf]

Supplementary Table S5. Associations between baseline inflammatory markers and longitudinal progression assessed using linear mixed-effects models adjusted for baseline age, sex, IOL, log-transformed EZ vertical length, CME and ERM and genotype group.

Outcome	Inflammatory marker	No. of patients	No. of observations	β for Month × Marker	95% CI	P value	FDR q value
ETDRS BCVA	Aqueous flare	67	594	-0.068	-0.145 to 0.009	0.083	0.203
ETDRS BCVA	IL-8	67	594	-0.004	-0.107 to 0.098	0.932	0.932
ETDRS BCVA	Intermediate monocyte	66	585	-0.158	-0.261 to -0.055	0.003	**0.018**
ETDRS BCVA	hs-CRP	67	594	-0.138	-0.237 to -0.039	0.007	**0.030**
MD	Aqueous flare	67	595	-0.026	-0.048 to -0.003	0.024	0.098
MD	IL-8	67	595	-0.028	-0.058 to 0.001	0.060	0.175
MD	Intermediate monocyte	66	586	-0.013	-0.044 to 0.017	0.387	0.563
MD	hs-CRP	67	595	0.002	-0.027 to 0.031	0.898	0.927
RS Cent 4'	Aqueous flare	67	595	-0.040	-0.069 to -0.011	0.006	**0.030**
RS Cent 4'	IL-8	67	595	-0.031	-0.070 to 0.007	0.112	0.257
RS Cent 4'	Intermediate monocyte	66	586	-0.004	-0.044 to 0.035	0.824	0.911
RS Cent 4'	hs-CRP	67	595	0.008	-0.030 to 0.046	0.683	0.841
RS Cent 1'	Aqueous flare	67	595	-0.034	-0.071 to 0.003	0.072	0.192
RS Cent 1'	IL-8	67	595	-0.009	-0.058 to 0.041	0.732	0.867
RS Cent 1'	Intermediate monocyte	66	586	0.006	-0.045 to 0.056	0.826	0.911

Outcome	Inflammatory marker	No. of patients	No. of observations	β for Month × Marker	95% CI	P value	FDR q value
RS Cent 1'	hs-CRP	67	595	0.028	-0.020 to 0.076	0.256	0.454
EZ vertical length	Aqueous flare	67	594	-0.002	-0.003 to -0.001	0.002	**0.016**
EZ vertical length	IL-8	67	594	-0.001	-0.003 to 0.001	0.277	0.467
EZ vertical length	Intermediate monocyte	66	585	-0.001	-0.003 to 0.001	0.188	0.375
EZ vertical length	hs-CRP	67	594	-0.001	-0.002 to 0.001	0.364	0.555
EZ horizontal length	Aqueous flare	67	594	-0.001	-0.003 to 0.000	0.052	0.175
EZ horizontal length	IL-8	67	594	-0.001	-0.003 to 0.001	0.503	0.644
EZ horizontal length	Intermediate monocyte	66	585	-0.001	-0.003 to 0.001	0.459	0.612
EZ horizontal length	hs-CRP	67	594	-0.001	-0.003 to 0.001	0.341	0.546
Central foveal thickness	Aqueous flare	67	595	0.123	-0.003 to 0.248	0.056	0.175
Central foveal thickness	IL-8	67	595	-0.113	-0.281 to 0.054	0.183	0.375
Central foveal thickness	Intermediate monocyte	66	586	0.013	-0.159 to 0.185	0.885	0.927
Central foveal thickness	hs-CRP	67	595	0.261	0.098 to 0.423	0.002	**0.016**
AF ring area	Aqueous flare	67	590	0.008	0.004 to 0.011	<0.001	**0.002**
AF ring area	IL-8	67	590	-0.002	-0.007 to 0.003	0.454	0.612
AF ring area	Intermediate monocyte	66	581	0.008	0.003 to 0.013	0.002	**0.016**
AF ring area	hs-CRP	67	590	0.003	-0.002 to 0.008	0.251	0.454

*Sensitivity model 2 was additionally adjusted for CME, ERM, and genotype group (EYS, USH2A, and Others). All models included fixed effects for time, baseline inflammatory marker level, and their interaction, with participant-specific random intercepts. The β coefficient represents the Month × Marker interaction term. FDR q values were calculated using the Benjamini–Hochberg procedure. Outcome measures: ETDRS BCVA (letters); MD (dB); RS Cent 4′ = central 12-point mean retinal sensitivity (dB); RS Cent 1′ = central 4-point mean retinal sensitivity (dB); EZ vertical length and EZ horizontal length [ln(mm + 1)]; Central foveal thickness (µm); AF ring area [$\ln(mm^2 + 1)$]. Inflammatory markers: Aqueous flare [ln(pc/ms + 0.1)]; IL-8 [ln(pg/mL + 0.1)]; Intermediate monocyte [ln(% + 0.1)]; hs-CRP [ln(mg/dL + 0.1)]. ETDRS = Early Treatment Diabetic Retinopathy Study; BCVA = best-corrected visual acuity; IOL = intraocular lens; EZ = ellipsoid zone; IL-8 = interleukin-8; hs-CRP = high-sensitivity C-reactive protein.
