# Supplementary material for: Inflammatory Markers and Disease Progression in Moderate-Stage Retinitis Pigmentosa: Longitudinal Analysis from the Retinitis Pigmentosa Progression and Inflammatory Marker Registry Study
Source: Ophthalmol Sci. 2026 Aug 25;6(11):101381. doi: 10.1016/j.xops.2026.101381 (PMC13634498; doi:10.1016/j.xops.2026.101381)
Supplement: Supplementary file 7 [file mmc7.pdf]

Supplementary Table S6. Associations between baseline inflammatory markers and longitudinal progression assessed using linear mixed-effects models adjusted for age, sex, and IOL, without adjustment for baseline log-transformed vertical EZ length in models of EZ length outcomes

Outcome	Inflammatory marker	No. of patients	No. of observations	β for Month × Marker	95% CI	P value	FDR q value
EZ vertical length	Aqueous flare	67	594	-0.002	-0.003 to -0.001	0.002	**0.013**
EZ vertical length	IL-8	67	594	-0.001	-0.003 to 0.001	0.272	0.495
EZ vertical length	Intermediate monocyte	66	585	-0.001	-0.003 to 0.001	0.188	0.495
EZ vertical length	hs-CRP	67	594	-0.001	-0.002 to 0.001	0.371	0.495
EZ horizontal length	Aqueous flare	67	594	-0.001	-0.003 to 0.000	0.052	0.208
EZ horizontal length	IL-8	67	594	-0.001	-0.003 to 0.001	0.502	0.502
EZ horizontal length	Intermediate monocyte	66	585	-0.001	-0.003 to 0.001	0.459	0.502
EZ horizontal length	hs-CRP	67	594	-0.001	-0.003 to 0.001	0.342	0.495

*Linear mixed-effects models included fixed effects for time, baseline inflammatory marker level, and their interaction, with participant-specific random intercepts. Models were adjusted for age, sex, and intraocular lens (IOL) status, but not for baseline ellipsoid zone (EZ) length. The β coefficient represents the Month × Marker interaction term. FDR q values were calculated using the Benjamini–Hochberg procedure. Outcome measures: EZ vertical length and EZ horizontal length [ln(mm + 1)]. Inflammatory markers: Aqueous flare [ln(pc/ms + 0.1)]; IL-8 [ln(pg/mL + 0.1)]; Intermediate monocyte [ln(% + 0.1)]; hs-CRP [ln(mg/dL + 0.1)]. EZ = ellipsoid zone; IOL = intraocular lens; IL-8 = interleukin-8; hs-CRP = high-sensitivity C-reactive protein.
