# Supplementary material for: Inflammatory Markers and Disease Progression in Moderate-Stage Retinitis Pigmentosa: Longitudinal Analysis from the Retinitis Pigmentosa Progression and Inflammatory Marker Registry Study
Source: Ophthalmol Sci. 2026 Aug 25;6(11):101381. doi: 10.1016/j.xops.2026.101381 (PMC13634498; doi:10.1016/j.xops.2026.101381)
Supplement: Supplementary file 8 [file mmc8.pdf]

Supplementary Table S7. Assessment of linearity over the 24-month follow-up using quadratic time terms in linear mixed-effects models.

Outcome	No. of patients	No. of observations	β for quadratic time term	95% CI	P value
ETDRS BCVA	67	594	0.177	-0.722 to 1.076	0.699
MD	67	595	-0.044	-0.306 to 0.217	0.739
RS Cent 4'	67	595	-0.030	-0.371 to 0.310	0.861
RS Cent 1'	67	595	0.168	-0.266 to 0.602	0.448
EZ vertical length	67	594	-0.003	-0.018 to 0.012	0.722
EZ horizontal length	67	594	0.008	-0.009 to 0.025	0.351
Central foveal thickness	67	595	-0.381	-1.854 to 1.092	0.611
AF ring area	67	590	-0.008	-0.051 to 0.036	0.731

* Quadratic time terms were added to the linear mixed-effects models to assess the assumption of linearity over the 24-month follow-up period. β represents the coefficient for the quadratic time term. No significant evidence of nonlinearity was observed for any outcome (all $P > 0.35$). Outcome measures: ETDRS BCVA (letters); MD (dB); RS Cent 4′ = central 12-point mean retinal sensitivity (dB); RS Cent 1′ = central 4-point mean retinal sensitivity (dB); EZ vertical length and EZ horizontal length [ln(mm + 1)]; Central foveal thickness (μm); AF ring area [ln(mm^2 + 1)]. ETDRS = Early Treatment Diabetic Retinopathy Study; BCVA = best-corrected visual acuity; EZ = ellipsoid zone.
